# Supplementary material for: Content-rich biological network constructed by mining PubMed abstracts
Source: BMC Bioinformatics. 2004 Oct 8;5:147. doi: 10.1186/1471-2105-5-147 (PMC528731; doi:10.1186/1471-2105-5-147)
Supplement: Additional File 2 — The original results of the above study (non-essential files are deleted to keep the file size under the limit set by BMC bioinformatics). [file 1471-2105-5-147-S2.bz2 › chilibotAdditionalFile2/dip30/156ID8438581E558/html/N_M2.html]

 


 **N** and **M2** 
  
Found 5408 abstracts in PubMed, retrieved 30.  
 

 What does Google say? 
 PDF only 
| .edu only 

---

**Interactive relationship** (e.g. stimulation, inhibition, etc)

**Non-interactive relationship** (e.g. studied together, co-existance, homology, etc.)

- Although cysteine substitution mutations to residues in the  **N**  terminal half of the alpha subunit  **M2**  M3 loop dramatically impaired the gating efficacy, the same mutations exerted little effect when incorporated into corresponding positions of the beta subunit.  Ref: 12871591 J Neurochem, 2003
- The filters were  **n** =10 PF1000N effective membrane surface 0.15  **m2** .  Ref: 12823420 Artif Organs, 2003
- 8 CdSO4 topology two new supramolecular isomers of  **M2**  bdc 2 L 2  **n**  coordination polymers.  Ref: 12841232 Chem Commun Camb , 2003
- Mean glomerular filtration rate decreased from 58 19 at 1 year  **n** =42 to 44 16 mL min 1.73  **m2**  body surface area at 10  **n** =33 years.  Ref: 12883184 Transplantation, 2003
- In addition, treatment with antioxidant  **N**  acetyl L cysteine inhibited anti Id induced apoptosis in G1 2 and  **M2**  10.  Ref: 12846046 Clin Exp Rheumatol< MedlineTA>Clin Exp Rheumatol, 2003
